# Supplementary material for: Nonproductive exposure of PBMCs to SARS‐CoV‐2 induces cell‐intrinsic innate immune responses
Source: Mol Syst Biol. 2022 Aug 17;18(8):e10961. doi: 10.15252/msb.202210961 (PMC9382356; doi:10.15252/msb.202210961)
Supplement: Supplementary file 1 — Appendix [file MSB-18-0-s003.pdf]

Table of Content

1. **Appendix Figure S1**. Remdesivir-sensitive SARS-CoV-2 infection of Vero E6 cells
2. **Appendix Figure S2**. Exposure of PBMCs to SARS-CoV-2 induced *IFIT1* mRNA expression in the absence of productive infection
3. **Appendix Figure S3**. Cytokine profile of SARS-CoV-. SARS-CoV-2- and mock-exposed PBMCs
4. **Appendix Figure S4**. Quality control metrics of scRNA-seq data
5. **Appendix Figure S5**. Ex vivo exposure with SARS-CoV or SARS-CoV-2 has no influence on T-cell activation and exhaustion
6. **Appendix Table S1**. Clinical features of COVID-19 patients from which sera were collected.

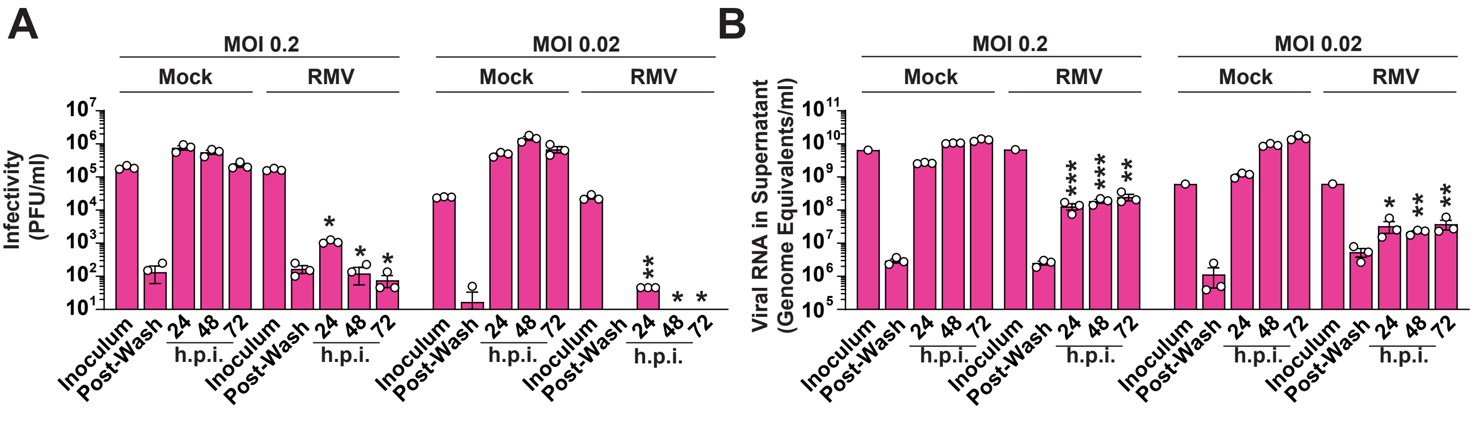

Appendix Figure S1. Remdesivir-sensitive SARS-CoV-2 infection of Vero E6 cells

Vero E6 cells were treated with Remdesivir (20 µM) or mock-treated and infected with SARS-CoV-2 at indicated MOIs. At indicated time points post-infection,
(A) infectivity in supernatant was quantified by plaque titration assays and
(B) viral RNA in supernatant was quantified by Q-RT-PCR.
Statistical significance was tested comparing Mock- to Remdesivir-treated samples using paired Student's t-test. Statistically significant differences were indicated as following: p value < 0.05 (*), p value < 0.01 (**) or p value < 0.001 (***). Symbols indicate cultures from three individual experiments, error bars indicate S.E.M. from four individual experiments. RMV = Remdesivir.

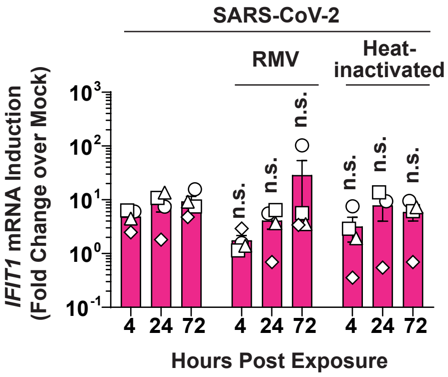

Appendix Figure S2. Exposure of PBMCs to SARS-CoV-2 induces *IFIT1* mRNA expression in the absence of productive infection

PBMCs were either mock-inoculated, pre-treated with Remdesivir or mock-treated, and challenged with SARS-CoV-2, or exposed to heat-inactivated SARS-CoV-2, and analyzed for *IFIT1* mRNA expression by Q-RT-PCR. Symbols indicate cultures from four individual donors, error bars indicate S.E.M. from four individual experiments.
Statistical significance was tested comparing mock- to Remdesivir-treated and heat-inactivated samples, respectively, using paired Student's t-test.
RMV = Remdesivir; n.s. = not significant

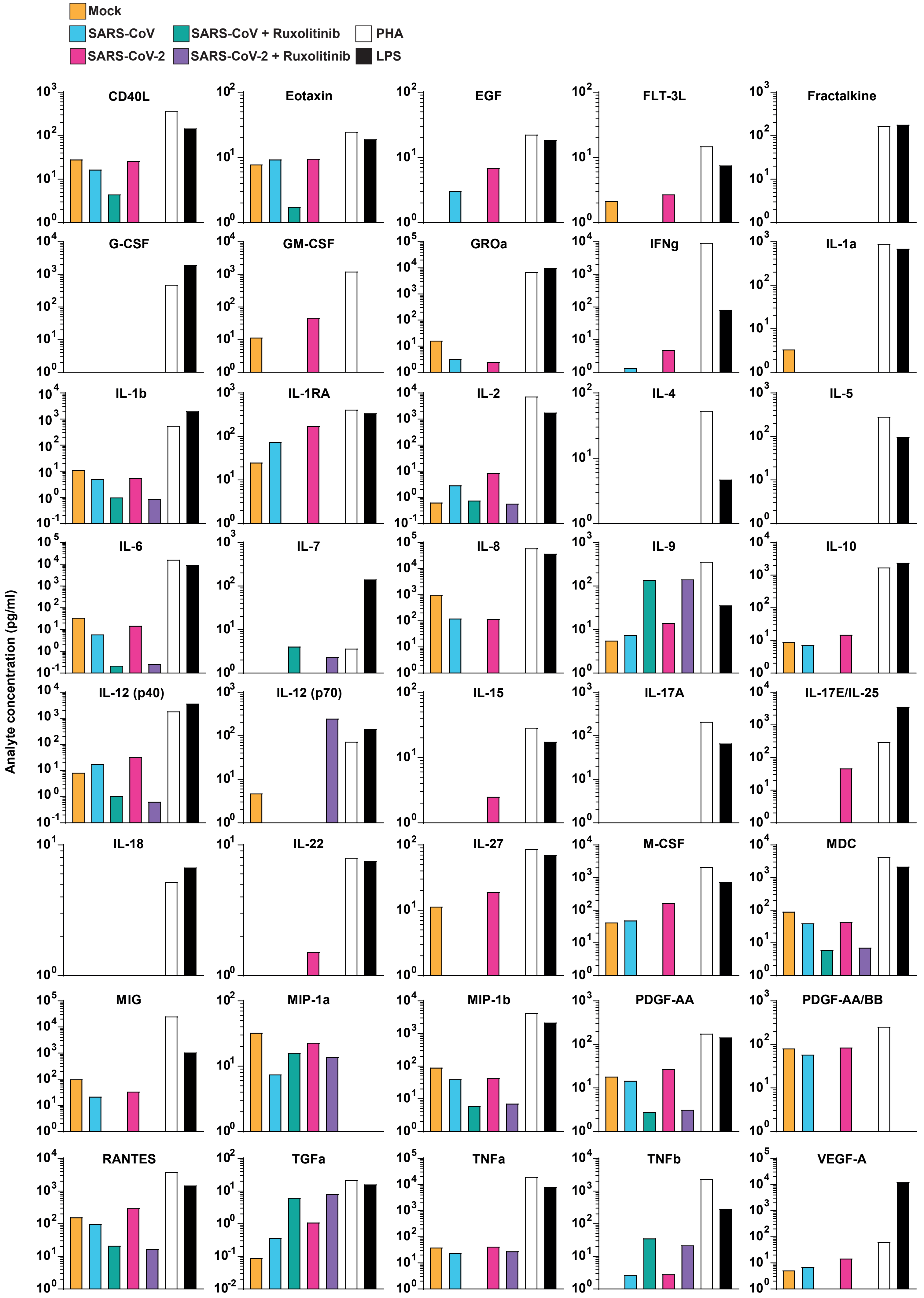
Mock
SARS-CoV
SARS-CoV + Ruxolitinib
PHA
SARS-CoV-2
SARS-CoV-2 + Ruxolitinib
LPS
Analyte concentration (pg/ml)
CD40L
Eotaxin
EGF
FLT-3L
Fractalkine
G-CSF
GM-CSF
GROa
IFNg
IL-1a
IL-1b
IL-1RA
IL-2
IL-4
IL-5
IL-6
IL-7
IL-8
IL-9
IL-10
IL-12 (p40)
IL-12 (p70)
IL-15
IL-17A
IL-17E/IL-25
IL-18
IL-22
IL-27
M-CSF
MDC
MIG
MIP-1a
MIP-1b
PDGF-AA
PDGF-AA/BB
RANTES
TGFa
TNFa
TNFb
VEGF-A

Appendix Figure S3. Cytokine profile of SARS-CoV-, SARS-CoV-2- and mock-exposed PBMCs

Indicated supernatants derived from four individual PBMC cultures from four donors were collected 48 hours post exposure, pooled, and cytokine protein expression was quantified using a Luminex-based immunoassay. PHA- and LPS-treated PBMC cultures were used as positive controls. Bars represent the individual measurements of a pool of four samples per condition.

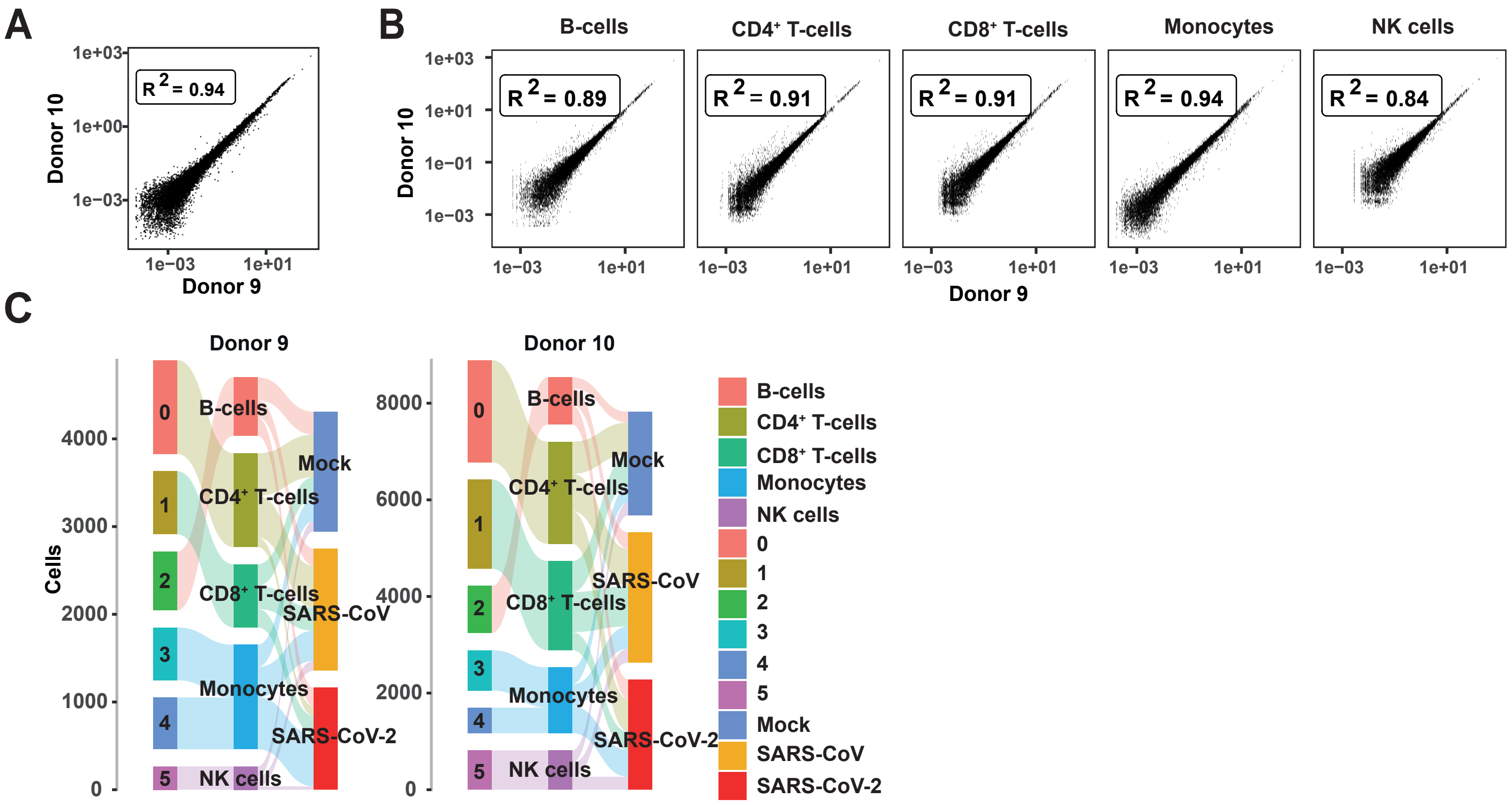

Appendix Figure S4. Quality control metrics of scRNA-seq data

Raw gene counts of
(A) all identified genes in donor #9 and #10 or
(B) separated by identified cell types.
(C) Graph showing the cell number distribution of each sample in Seurat cluster (left), cell type annotations (middle) and treatments (right).

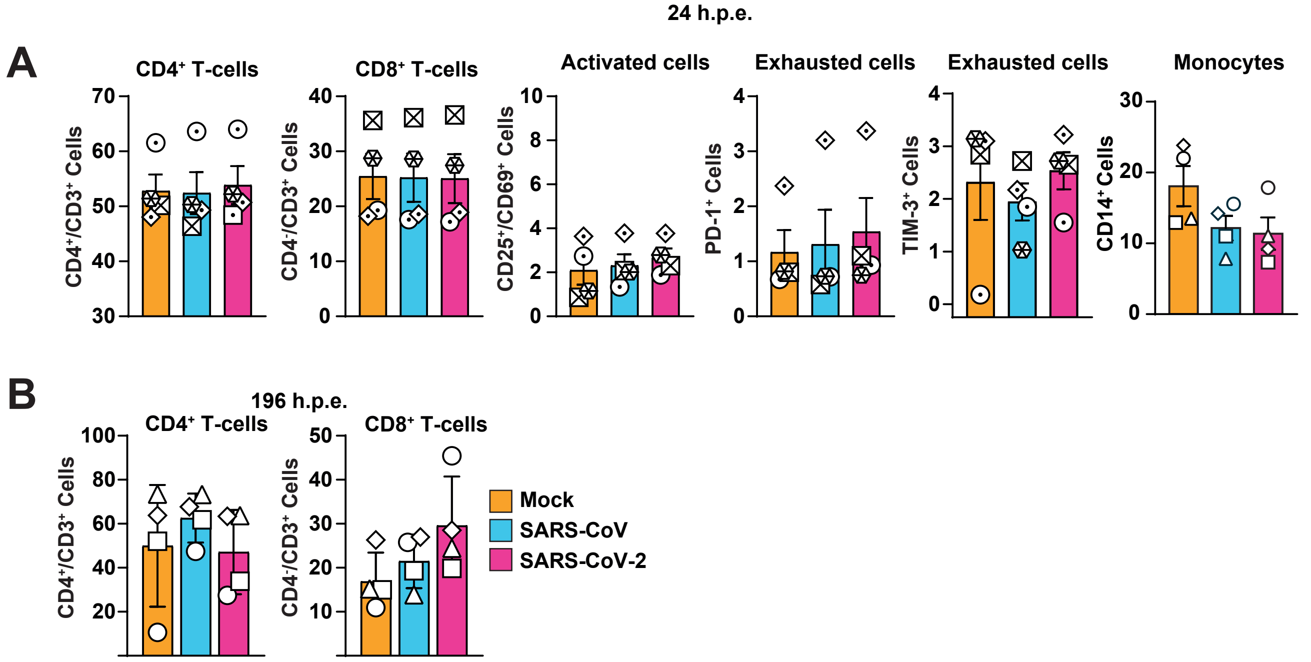

Appendix Figure S5. *Ex vivo* exposure with SARS-CoV or SARS-CoV-2 has no influence on T-cell activation and exhaustion

Surface immunostaining of SARS-CoV or SARS-CoV-2-exposed or mock-exposed PBMCs for T cell surface marker CD3, CD4, CD25/CD69, PD-1 and TIM-3 **(A)** 24 hours post-exposure or **(B)** 192 hours post-exposure. Symbols indicate cultures from four individual donors, error bars indicate S.E.M. from four individual experiments.

Donor ID	Max. WHO Disease Severity Score	WHO Disease Severity Score	Days post Symptom Onset	CT Value (Swab Samples)
1	3	3	16	14.80
2	3	3	8	38.64*
3	3	3	11	32.83

Appendix Table 1. Clinical features of COVID-19 patients from which sera were collected

Clinical characteristics of serum samples used in this study. *Oropharyngeal swab was taken one day post serum sampling.
