# Supplementary material for: Nonproductive exposure of PBMCs to SARS‐CoV‐2 induces cell‐intrinsic innate immune responses
Source: Mol Syst Biol. 2022 Aug 17;18(8):e10961. doi: 10.15252/msb.202210961 (PMC9382356; doi:10.15252/msb.202210961)
Supplement: Supplementary file 2 — Expanded View Figures PDF [file MSB-18-0-s001.pdf]

Expanded View Figures

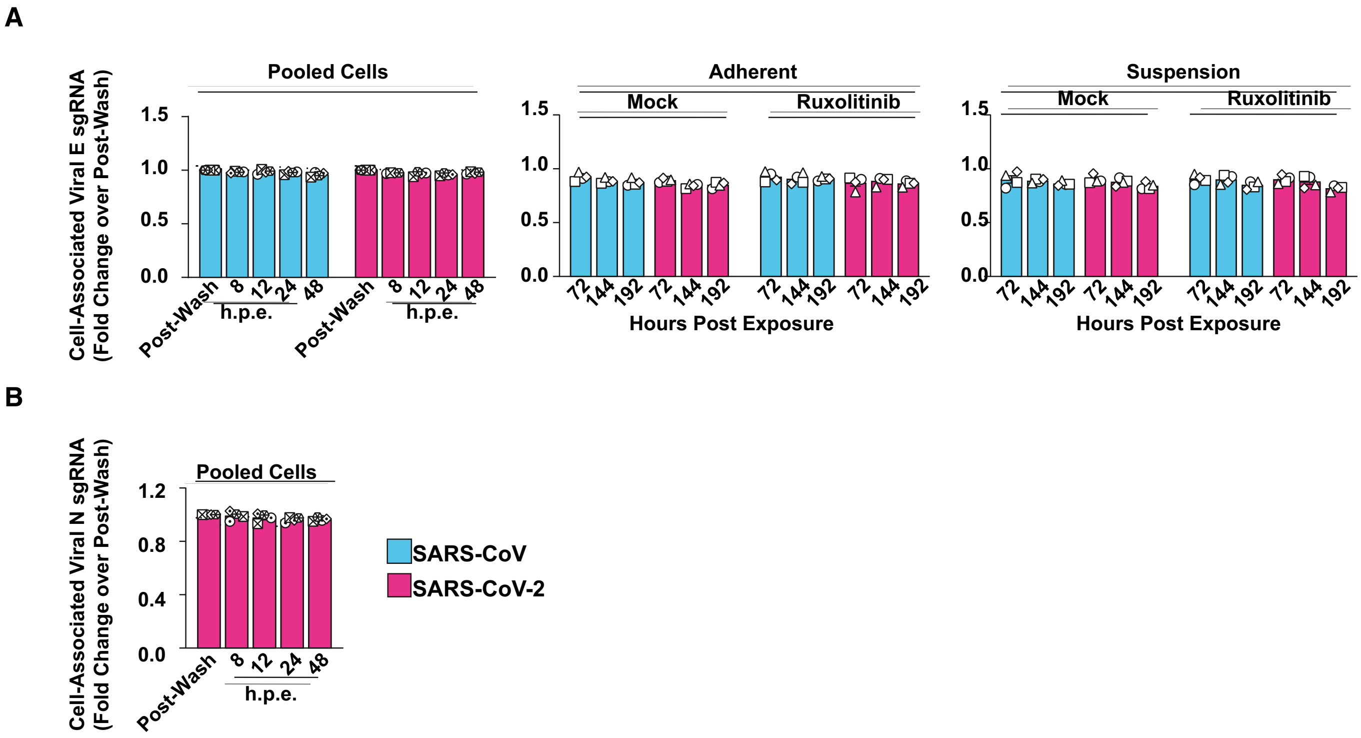

Figure EV1. Absence of *de novo* production of subgenomic viral RNA in SARS-CoV- and SARS-CoV-2-inoculated PBMCs.

PBMCs were treated with Ruxolitinib (10 μM) or mock-treated and challenged with SARS-CoV, SARS-CoV-2, or mock-challenged.

A, B Cell-associated subgenomic viral RNA (sgRNA encoding envelope (E) gene, (A)) or nucleocapsid (N) gene, (B)) at indicated time points was quantified in pooled, suspension, and adherent cells by Q-RT–PCR and normalized to postwash samples. Symbols indicate cultures from four individual donors; error bars indicate SEM from four individual experiments.

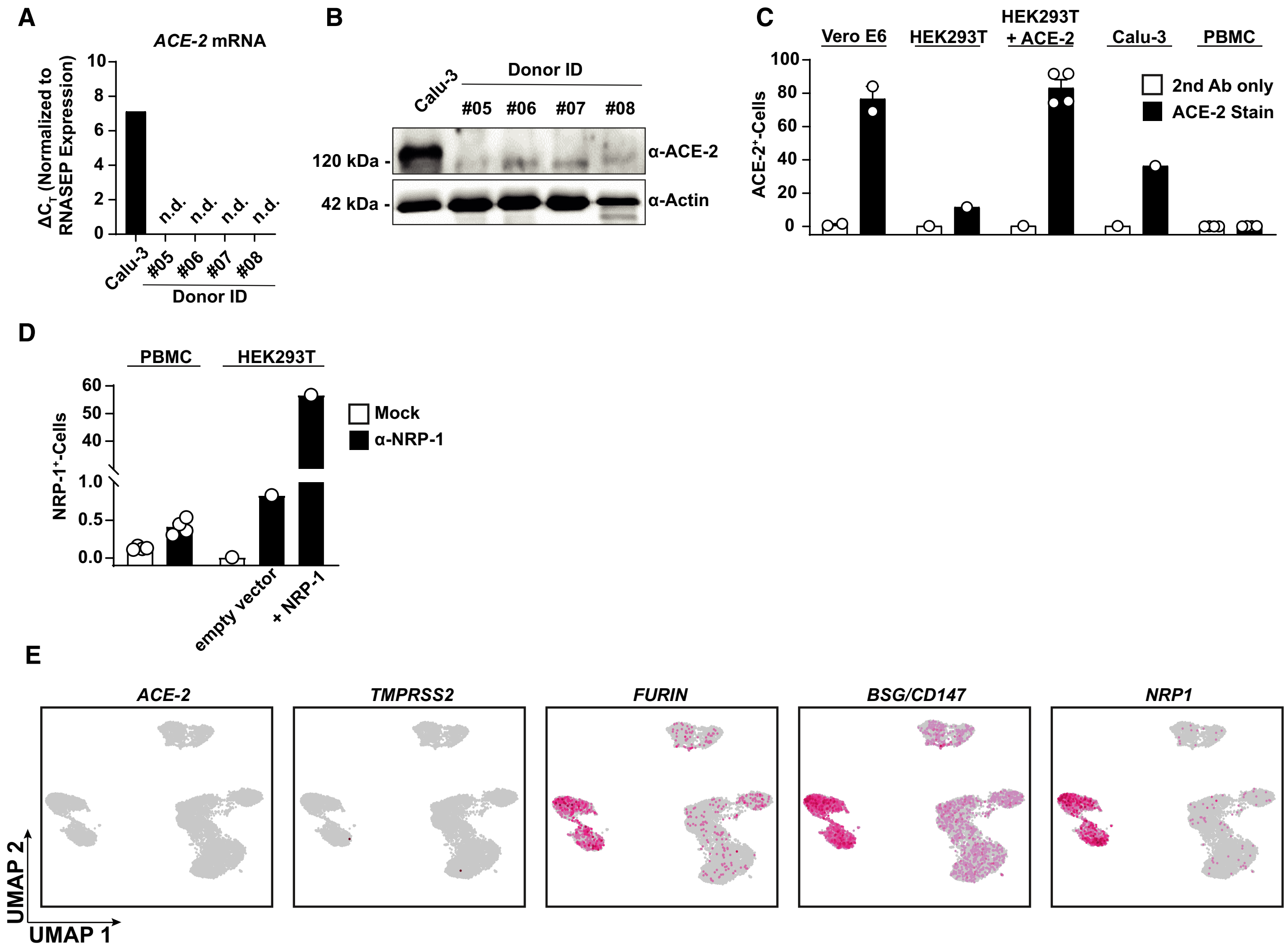

Figure EV2. Cofactor expression profile in PBMCs.

A–C ACE2 expression was analyzed in PBMC lysates from four biological replicates and Calu-3 cells by Q-RT–PCR of *ACE2* mRNA (A), anti-ACE2 immunoblotting (B), and anti-ACE2 immunostaining followed by flow cytometric analysis (C). Bars represent the mean, error bars indicate the SEM.

D Surface NRP1 expression was quantified by flow cytometry in PBMCs and indicated HEK293T cells. Symbols indicate cultures from four biological replicates (PBMCs) or HEK293T cells ($n = 1$); error bars indicate SEM.

E UMAPs showing expression of *ACE2*, *TMPRSS2*, *FURIN*, *BSG*, and *NRP1* in all analyzed cells. Data shown are based on the analysis of two donors.

© 2022 The Authors

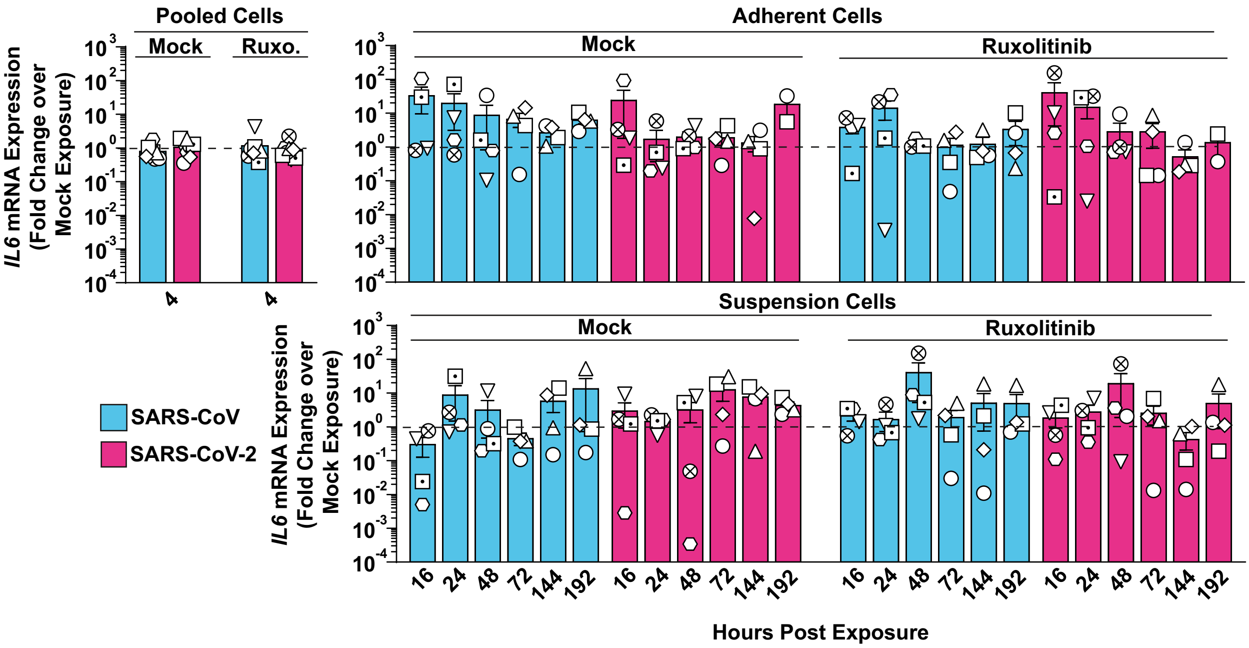

Figure EV3. SARS-CoV and SARS-CoV-2 exposure does not trigger an *IL6* response in PBMCs.

RNA extracted from Ruxolitinib-treated or mock-treated, and SARS-CoV-, SARS-CoV-2-, or mock-exposed PBMCs was analyzed for *IL6* mRNA expression by Q-RT–PCR at indicated time points. Suspension and adherent cell fractions were analyzed separately, except at the 4 h time point. Values were normalized to cellular *RNASEP* expression and are shown as fold change over mock-exposed conditions. The dotted line indicates the expression level of mock-inoculated cell cultures and is set to 1. Symbols indicate cultures from four individual donors; error bars indicate SEM from four individual experiments.

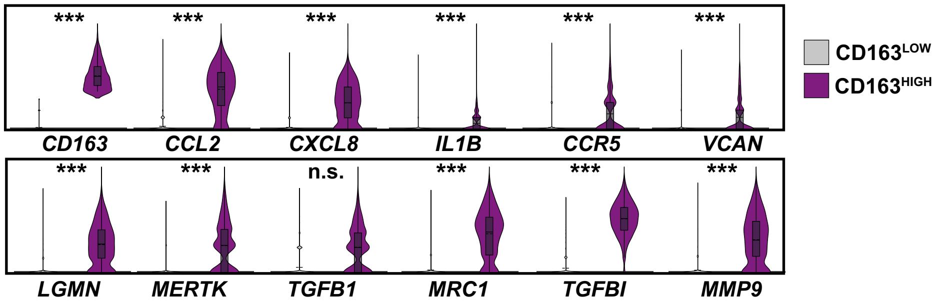

Figure EV4. CD163^{HIGH} monocytes associate with marker genes specific for fibrosis.

Distribution of indicated gene expression in CD163^{HIGH} and CD163^{LOW} monocytes (425 and 718 cells, respectively). Cells were considered as CD163^{HIGH} with a Log_2(CD163 average expression) > 2. A total of 1,520 CD163^{HIGH} and 20,962 CD163^{LOW} cells were analyzed. Data shown in this figure are based on the analysis of two donors. Statistical significance was tested with the negative binomial exact test; *P*-values < 0.05 were considered significant and marked accordingly: $P < 0.05$ (*), and $P < 0.01$ very significant (**), or $P < 0.001$; n.s. = not significant (≥ 0.05).

© 2022 The Authors

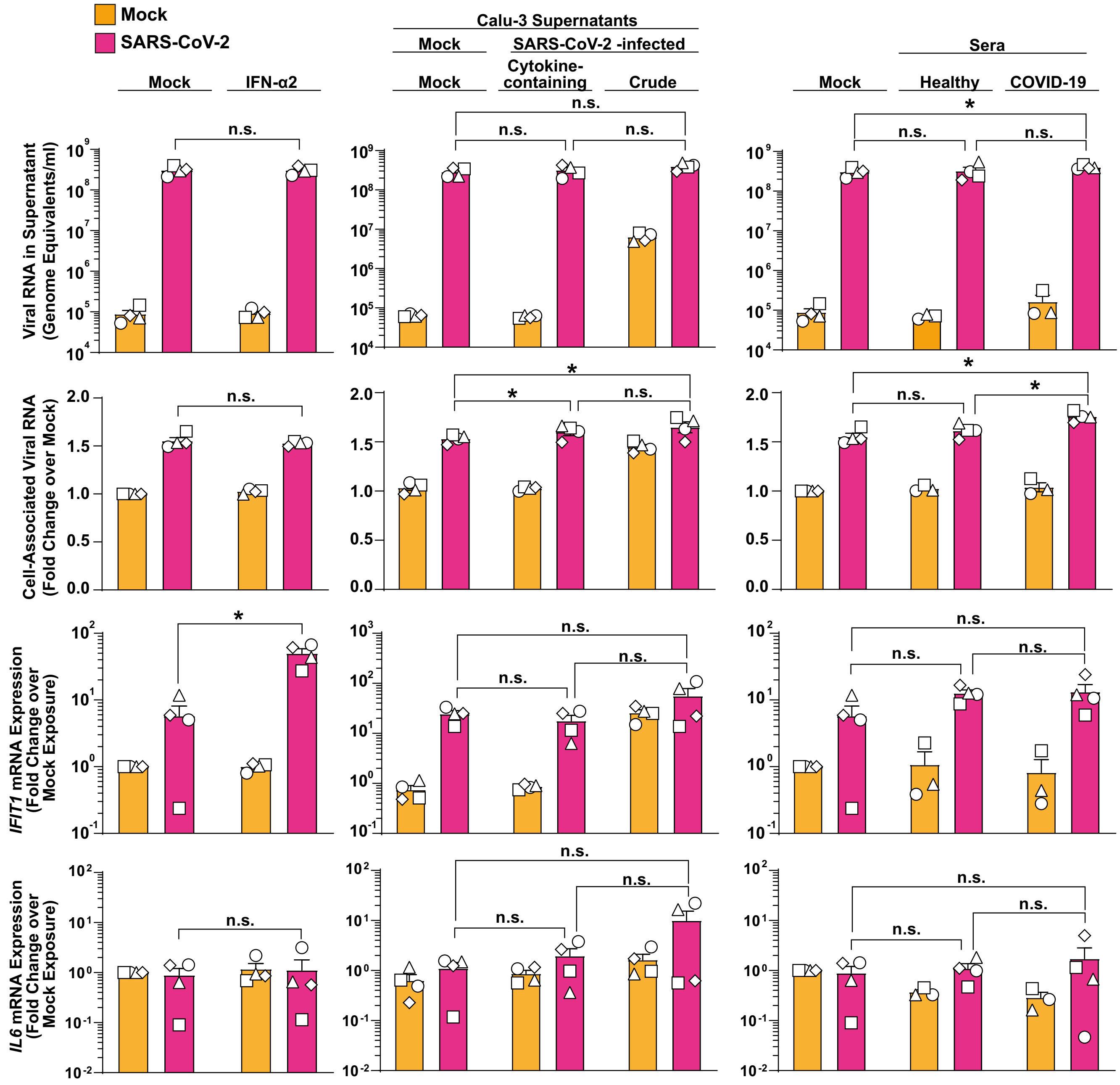

Figure EV5. PBMCs are sensitized for virus uptake upon pretreatment with SARS-CoV-2-induced cytokines.

PBMCs were preincubated with 100 IU/ml IFN-a2a (left panels), supernatants from mock-treated Calu-3, nonfiltered or filtered, cytokine-containing, and virus-free supernatants from SARS-CoV-2 infected Calu-3 (middle panels) or sera from healthy controls and mildly diseased COVID-19 patients for 18 h before exposure to SARS-CoV-2 for another 24 h. Cultures were analyzed for:

A Viral RNA concentrations (genome equivalents/ml) in cell-culture supernatants by Q-RT–PCR.
B Relative changes of cell-associated viral genomic RNA quantities by Q-RT–PCR.
C *IFIT1* mRNA expression by Q-RT–PCR.
D *IL6* mRNA expression by Q-RT–PCR.

Data information: Statistical significance was tested using the paired Student's *t*-test comparison. *P*-values > 0.05 were considered not significant (n.s.), *P*-values < 0.05 were considered significant (*), and *P*-values < 0.01 were considered very significant (**). Symbols indicate cultures from four individual donors; error bars indicate SEM from four individual experiments.

 © 2022 The Authors
